# Research priorities set by people with OCD and OCD researchers: Do the commonalities outweigh the differences?

**DOI:** 10.1111/hex.13005

**Published:** 2019-11-28

**Authors:** Franziska Kühne, Anna Levke Brütt, Mara Jasmin Otterbeck, Florian Weck

**Affiliations:** ^1^ Department of Psychology, Clinical Psychology and Psychotherapy University of Potsdam Potsdam Germany; ^2^ Department for Health Services Research Carl von Ossietzky University Oldenburg Oldenburg Germany

**Keywords:** clinical psychology, mental disorders, obsessive‐compulsive disorder, patient empowerment, patient involvement, Patient participation

## Abstract

**Objective:**

In contrast to research agendas being predominantly set by scientists or funders, a collaborative approach was used to spot future goals for research on obsessive‐compulsive disorder.

**Methods:**

First, we conducted a meta‐review and then compared the results of two online surveys with OCD professionals and patients on research priorities. The literature search was performed in three comprehensive databases, and ten research goals were extracted. Sixty‐four patients and eight professionals responded to open questions on their five most important goals. Then, they ranked the ten aims extracted from the literature on a 6‐point Likert scale.

**Results:**

For patients and professionals, research on treatment gains that persist long‐term was most important. Concerning the top five goals listed in an open format, for patients, development and maintenance of the disease was as important as psychotherapy and its efficacy. In contrast, for professionals, the efficacy and the optimization of psychotherapy were the far most important research goals.

**Conclusions:**

We proposed one possibility to involve patients in OCD research, and the multitude of answers presents a wealth of research ideas.

**Practice Implications:**

Since consistent research involvement may contribute to its clinical impact, researchers are now invited to translate our findings into empirical studies.

## 1 INTRODUCTION

Patient involvement (PI) may be described as the participation of patients or users based on their individual experience with a disease or with specific treatments.^1^ Tritter^2^ characterizes the following types of PI: participation in treatment decisions, in service development and the evaluation of services, in education and training, and in research activities. Participation is generally indicated as an active and collaborative process,^3^ and involvement into research gives credit to patients’ perspectives, interests and values.^4^ For example, it aims at asking meaningful research questions, improving study feasibility, supporting the dissemination of relevant findings into clinical practice or improving health outcomes.^4^, ^5^ Against this backdrop, PI was acknowledged by the Lancet Psychiatry Commission on Psychological Treatments Research as one means for advancing psychotherapy research.^6^

The scope of PI varies considerably, from organizational involvement to writing lay summaries, from single to continuous participation.^4^ Considering Farin‐Glattacker and colleagues’^7^ matrix, we aimed at early involvement and at equal collaboration; that is, in our study, research priorities will be identified equally by researchers and patients, and patients will have the final say. This is also in line with results of a former own study, in which some patients encouraged earlier involvement, especially as to defining the research agenda.^4^

One of the most prominent associations asking patients to prioritize research goals is the James Lind Alliance, which also published a guidebook to enhance identifying the ten most important research aims from patients, carers and professionals, also to inform funding agencies.^8^ Although we had to align to the resources available within our institutions, we still got inspired by their inclusive, transparent and evidence‐based approach.

Former PI studies in our field referred to research priorities in mental health in general^9^ or to uncertainties in the treatment of specific subgroups, such as patients diagnosed with Parkinson's disease.^10^ Regarding mental health, during the online survey conducted by Haarig and colleagues,^11^ patients diagnosed with bipolar disorder were asked for the individually most important therapeutic goals. Coping with the symptoms of the disease was most important to the participants, and clearly inferior were improvement of quality of life, participation in psychotherapy itself and the management of adverse effects of the medication.^11^ Another study focused on research questions of patients with depression.^12^

To our knowledge, there is no PI study specifically on the psychotherapy for obsessive‐compulsive disorder (OCD) and respective research priorities to date. Analysing former studies, Ennis & Wykes^5^ pointed out that studies on difficult‐to‐treat populations such as patients diagnosed with developmental and personality disorders were associated with lower PI. Similar prejudices seem to persist on OCD, for example, that patients were rather difficult to treat,^13^ which could also explain the scarcity of PI in this field. OCD is characterized by recurrent and persistent thoughts, urges or images experienced as intrusive and unwanted causing anxiety or distress (obsessions), and consequently, repetitive behaviours or mental acts to reduce discomfort (compulsions).^14^ Whereas untreated OCD tends to be a rather chronic disease,^15^, ^16^ a number of patients are not reached by existing evidence‐based approaches, namely cognitive‐behavioural therapy (CBT) or selective serotonin reuptake inhibitors (SSRI), and therapists do not always implement CBT as intended.^16^, ^17^, ^18^ Although therapy improved during the last decades, relapse rates are still unsatisfactory.^16^ Since there is still room for improvement in the psychotherapy for OCD,^19^ PI has the potential to contribute to a meaningful research agenda. Further, at least in Germany, PI into psychotherapy research is still in its infancy. Furthermore, current therapy is stretched to its limits as prominent emotions in OCD, such as disgust, tend to habituate more slowly than anxiety, which may impair the efficacy of therapy if not considered sufficiently.^20^ As outlined above, OCD differs from other anxiety disorders as it is still considered difficult to treat, due to other susceptibilities to change, through less appropriate care, and thus higher relapse rates and chronic trajectories. With that said, the aim was to spot future goals for research specifically on obsessive‐compulsive disorder (OCD). Therefore, we examined the current literature through a meta‐review and surveyed professionals (Study 1) and patients (Study 2) with regard to psychotherapy and OCD‐related research priorities. Subsequently, we compared patients’ and professionals’ views in order to examine commonalities and differences and, thus, to support patient‐focused research agendas.

## 2 METHODS

To derive research aims from the current literature, three databases (the International Prospective Register of Systematic Reviews (Prospero), the Cochrane Database of Systematic Reviews (CDSR) and the Campbell Collaboration) were searched in June 2017 for reviews on obsessive‐compulsive and related disorders published between 2002 and 2017. From n = 47 records without duplicates screened by title and abstract, n = 34 were excluded due to different reasons (no adult population (n = 15), review uncompleted (n = 12), no OCD (n = 7)); thus, n = 13 reviews were included from that search. Searching the references of the German S3‐Guideline on Obsessive‐Compulsive Disorders^17^ resulted in n = 6 additional reviews, so that N = 19 reviews were included in the meta‐study. From these reviews (see Appendix S1), the top ten aims for future research on the psychotherapy of OCD were extracted from the discussion sections by one researcher (xx), discussed with another (xx), and then phrased as concise items for the subsequent surveys (Studies 2 and 3).

### 2.1 Study 1: professionals’ survey

In November 2017, national professionals in the field of OCD research and treatment were contacted via e‐mail and asked for participation in the online survey, or asked for forwarding the invitation to other experts. Interested professionals were redirected to the survey implemented via UP Survey, a protected web‐based survey offered by the University of Potsdam. The first section of the survey comprised the study description and sociodemographic questions. Two open questions to name and rank the five individually most important aims for OCD research in general and research on psychotherapy with OCD patients in particular followed. Then, professionals were presented with the ten research aims extracted from the meta‐review (Study 1) and asked to rate the priority of each on a 6‐point Likert scale from very unimportant (1) to very important (6). Altogether, eight professionals participated (Table 1).

**Table 1:** Demographic data (*n* if not otherwise specified)

Patients (N = 63)	
Age^a^	37.8 (12.8, 18‐70)
Gender (female)	47 (74.6%)
Education	
≤10 y	16 (25%)
>10 y	47 (75%)
First OCD diagnosis^a^	2004 (11.1, 1969‐2018)
Current treatment^b^	28 (44.4%)
Cognitive‐behavioural	19
Medication	17
Psychodynamic	4
Prior treatment^b^	48 (76.2%)
Cognitive‐behavioural	40
Medication	27
Psychodynamic	17
Current and prior treatment	18 (28.6%)

Professionals (N = 8)	
Profession	
Psychologist	6
Physician	1
Other	1
Professional years^a^	
	14.9 (9.6, 0‐29)
Degree	
Licensed	6
MSc diploma	1
Other	1
Scope of work^b^, ^c^	
Patient care	40% (5%‐80%)
Research	30% (0%‐75%)
Teaching	13% (0%‐30%)
Others	20% (0%‐40%)

^a^ Mean (SD, range).

^b^ Multiple indications possible.

^c^ Mean % (range).

### 2.2 Study 2: patient survey

To anonymously gather data on OCD patients’ perspectives on psychotherapy research, we again conducted an independent Internet‐based survey. Participants’ health expertise was addressed, and they were invited online (via the German Society for Obsessive‐Compulsive Disorders (DGZ), a self‐help website and our department's website. Interested patients were again forwarded to the survey implemented via UP Survey. Recruitment proceeded from January 2018 to January 2019. Only adult patients who indicated that their OCD diagnosis had been established by a physician or psychologist were included. No monetary compensation was offered.

The survey contained the study description, an electronic informed consent, sociodemographic questions, and questions regarding pre‐experience with psychotherapy. Then, patients were asked with an open question to name and rank the five individually most important aims for psychotherapy research on OCD. Like the experts, they were then presented with the ten research aims extracted from the meta‐review and asked to rate the priority of each on the 6‐point Likert scale. The ethical aspects of study 3 were approved by the University of Potsdam ethics review board (no. 9/2017).

### 2.3 Data analysis

Due to the different sample sizes between professionals and patients, we mainly used descriptive statistics. According to the means, standard deviations and ranges, the items were ranked in descending order and independently for patients and professionals. In line with Banfield et al^9^ and due to skewness, we also dichotomized the scale (ie combined important and very important ratings versus all others) and ranked the items again. Due to the data structure and unequal sample sizes, we then examined differences between the central tendencies of patients and professionals via a non‐parametric Mann‐Whitney *U*‐test, that is, one analysis over the 10 goals. To determine agreement among participants regarding the 10 goals, we used Krippendorff's α, a flexible reliability measure considering any number of categories, any number of judges and missing data,^21^, ^22^ that is implemented within an SPSS macro.^23^ Krippendorff's *α* = 1 presents perfect agreement, whereas *α* = 0 defines its absence.^22^ All analyses were performed using IBM SPSS Statistics 25 and Microsoft Excel at a 0.05 level of significance.

Open questions regarding the top 5 research priorities were analysed qualitatively using inductive content analysis techniques.^24^ First, three researchers (xx, xx, xx) read all answers to familiarize with the data. Preliminary categories were developed separately for patients and professionals and then discussed to foster a common understanding. Following this, the final categories were derived by two independent researchers (xx, xx/xx). Inter‐rater agreement reached *κ* = 0.81 (patients’ category system) resp. *κ* = 0.92 (professionals’ category system). To generate a hierarchy, the priorities given by the participants in the quantitative part were inverted (ie the individual priority 1 was inverted to 5 to give it the most weight) and summed up within each category. Then, for indications and priorities, their number per category was divided by their total number in order to obtain scores comparable between the two groups. Since for professionals, very similar qualitative answers were given with respect to both research questions (top 5 aims for OCD research in general and for research on psychotherapy with OCD patients in particular), we summarized the two into a joint category system. In the subsequent patient survey, we therefore focused on the psychotherapy question only.

## 3 RESULTS

### 3.1 Goals from the meta‐review

The research goals extracted from the current literature (Table 2, Appendix S1) concerned the following overarching topics: comparative effectiveness, mechanisms, moderators, administration, target groups, motivation, dissemination, quality of life and treatment gains that persist long‐term.

**Table 2:** Prioritized research goals (from 1 = very unimportant to 6 = very important; patients’ ranges were 1 ‐ 6 for all items)

Goals from meta‐review	Patients			Professionals		
	M (SD) Rank	(very) important Rank	n	M (SD); range Rank	(very) important Rank	n
Which factors influence the long‐term effectiveness of PT? (goal #7)	5.3 (1.2) **1**	78% **1**	59	5.7 (0.5); 5‐6 **1**	100% **1**	8
How effective are PT approaches in comparison (eg CBT vs. psychodynamic vs. client‐centred)? (goal #1)	5.0 (1.3) **2**	71% **2**	63	4.5 (2); 1‐6 **9**	63% **4**	8
How to disseminate effective treatments into practice? (goal #9)	5.0 (1.3) **2**	71% **2**	60	5.3 (0.8); 4‐6 **4**	75% **3**	7
How do different characteristics influence the efficacy of PT (eg duration, frequency, role of therapist)? (goal #2)	4.9 (1.3) **3**	65% **5**	63	5.0 (1.2); 3‐6 **7**	63% **4**	8
By which means is PT effective (therapy mechanisms, eg habituation)? (goal #6)	4.8 (1.3) **4**	70% **3**	62	5.4 (0.5); 5‐6 **2**	100% **1**	8
How effective is PT including relatives (family, friends)? (goal #10)	4.7 (1.4) **5**	60% **6**	62	5.4 (0.7); 4‐6 **3**	88% **2**	8
How does PT impact on patients’ quality of life? (goal #3)	4.7 (1.4) **5**	67% **4**	63	4.7 (1.0); 3‐6 **8**	63% **4**	8
How to motivate patients for confrontation therapy? (goal #8)	4.6 (1.5) **6**	57% **7**	62	5.3 (1.2); 3‐6 **5**	75% **3**	8
How effective is online PT (eg Internet‐based CBT supported by a therapist)? (goal #4)	4.0 (1.7) **7**	44% **8**	61	5.0 (0.8); 4‐6 **6**	75% **3**	8
How effective is PT delivered in groups vs. individually? (goal #5)	3.7 (1.4) **8**	30% **9**	63	4.1 (1.3); 2‐6 **10**	38% **5**	8

Abbreviations: CBT, cognitive‐behavioural therapy; PT, psychotherapy. Ranks are prinited in bold.

### 3.2 Quantitative ranking results

A total of 150 persons followed the link, and of these, N = 63 were included in the final analyses (Table 1). The others were excluded because they did not give electronic informed consent (n = 22), were younger than 18 years (n = 5), did not proceed until the ranking questions (n = 59), or filled in the questionnaire twice (n = 1).

Patients’ (N = 63) age varied from 18 to 70 years, and their age mean was 38 (SD = 13) years (Table 1). Most participants (75%) were female, well‐educated and had rather long disease and treatment experiences. Most were treated with CBT (30%) or medication (27%). The professionals (N = 8) were mainly experienced psychologists and licensed psychotherapists practicing research, teaching and patient care.

According to the quantitative rankings of the 10 predefined aims by patients and professionals, the most prominent goal was doing research ensuring treatment gains that persist long‐term (goal 7, Table 2). Both groups also agreed on the least important aim for future research, that is, the effectiveness of group versus individual psychotherapy (goal 5). For OCD patients, the comparative effectiveness of psychotherapy approaches (goal 1) and the dissemination of effective treatments into practice (goal 9) were mutually important. Still, there was less agreement among the two groups on the other research goals. Concerning ranges and percentage‐wise agreement on importance, professionals evaluated the given goals more consistently than the patients did, but this may also be attributable to small sample size. For the ten research goals, differences in the central tendencies between the two groups were non‐significant (*P*>.05); that is, patients and professionals evaluated every goal as comparably important. Neither was there agreement among the patients (Krippendorff's *α* = .097) nor among the professionals (Krippendorff's *α* = .124) regarding prioritization of the 10 predefined goals.

### 3.3 Qualitatively found priorities

Considering the open answers, that is, to name the five individually most important aims for psychotherapy in OCD, six (patients) and five (professionals) relatively comparable categories emerged (Table 3). Most often, patients indicated aspects that fell within the category ‘Disease development and maintenance’. Example statements were ‘What is the cause for OCD, and how should psychotherapy that is related to the causes look like? [P22]’ or ‘What's going on in the body? [P37]’. Second most often, patients were asking for research on ‘Psychotherapy and its efficacy’, for example, the indication for different therapeutic approaches especially for exposure, its alternatives, for group and in‐patient therapy or treatments whose gains persist long‐term. At a distance, questions regarding the ‘Course of the disease’ were mentioned, and concerned for example the probabilities for complete recovery, of relapses or of exacerbation. Fourth, although not in the focus of the survey, patients asked for more effective ‘Psychopharmacotherapy’ with less side effects. Concerning the category ‘Improving the quality of care’, patients mentioned aspects such as ‘Why is there still a deficit in OCD treatment (too few experts, too long waiting times)? [P116]’. The last category (‘Others’) was mainly comprised of therapy‐related questions and of questions concerning self‐help, but also of research‐related criticism (eg ‘Why is so little research conducted on OCD? [P20]’ or ‘Why is there so little progress in OCD research? [P15]’; Appendix S2).

The professionals’ qualitative answers far most often fell within the category ‘Psychotherapy and its efficacy’. Example items were ‘What is effective psychotherapy for OCD? [E4]’, ‘How can we help patients (therapy resistant) who do not benefit from standard therapy (ERP)? [E2]’, or referred to motivation, nonresponse, differential indication, dismantling or the active ingredients of therapy. The second category concerned ‘Optimizing existing therapies’ but also referred to developing new approaches (such as extinction learning, reduction of subjective units of distress during exposure, virtual reality or the combination with medical therapies). Third were questions on ‘Disease development and maintenance’ (eg biographical and neurobiological factors), and fourth ‘Improving the quality of care’ (eg dissemination of effective therapies into health care, improving therapists’ willingness to treat OCD, expanding professional networks, reducing waiting lists for therapy). The last category comprised questions on OCD subtypes and on related disorders like trichotillomania (Appendix S3).

## 4 DISCUSSION AND CONCLUSION

### 4.1 Discussion

In order to examine priorities for future OCD research, we conducted a meta‐review and two subsequent online surveys with OCD patients and professionals. As one of the first OCD studies, we involved patients and combined quantitative and qualitative data. Whereas the development and maintenance of OCD and more therapy‐related questions were central to patients, professionals prioritized future research on the efficacy and optimization of psychotherapy. Both patients and professionals indicated treatment gains that persist long‐term as the most important research goal. In contrast, most current studies on psychotherapy and medication in OCD make use of short‐term durations of approximately 12 weeks^25^ or of on average 15‐month follow‐ups.^15^ Since OCD is often, at least without treatment, proceeding chronically,^18^ naturalistic research is on the one hand clearly necessary, but on the other complex and expensive. As Hansen and colleagues^26^ pointed out, so far only three trials examining exposure and response prevention (ERP) in OCD used follow‐ups of 24 months or longer, and all of them had severe problems with dropout. According to their analysis of ten trials, only 41% of OCD patients showed clinically significant improvement of OCD symptoms following ERP at post‐treatment and at on average 25 months of follow‐up. They conclude that ‘the question of what predicts long‐term outcome is basically not investigated’.^26^ ^(p91)^

Furthermore, patients probably consider different indicators of treatment success than professionals. In one of our own studies,^4^ patients indicated increased autonomy (eg larger scopes of action), the applicability of an intervention (eg in everyday life or in crises), better self‐perception (eg coping with oneself) and empowerment (ie knowing what helps oneself) as important treatment outcomes. Interview studies or focus groups would be appropriate to follow these issues.

Regarding the other research goals, there was low agreement, both within and between patients and professionals. Still, most goals were assessed as highly relevant by both groups underscoring the findings from the meta‐review. There were no significant differences between the means and ranks of the two groups which again indicate the comparable importance to patients and experts. Nevertheless, the patients’ rankings were distributed a bit more which may be attributable to more scepticism or to more problems with evaluating the relevance of every aspect. Subsequent studies using a forced choice approach may yield clearer results.^9^

Associated with their roles, in the open format, patients listed goals that were more therapy‐related, and professionals placed more emphasis on advancements in psychotherapy research. Research trainings adapted to patient participants are a feasible method to enable them to overlook the scope of research.^4^ As the variety of the 650 individual aspects listed by the patients accessible via the Supplements demonstrates, PI has the potential to direct our attention to clinically relevant topics. Beyond, PI enhances the understandability of study information and materials, the feasibility and acceptability of study designs and the commitment of patients if they know that other patients were previously involved.^5^, ^27^

Research on specific symptoms of the disease was more relevant to patients diagnosed with Parkinson's (eg balance problems, dyskinesia or cognitive problems)^10^ than for the OCD patients involved in our survey. In the future, studies could target the heterogeneous subgroups of OCD (such as contamination, harming, symmetry/ordering, pure obsessions)^20^ to clarify differential needs. Furthermore, patients diagnosed with depressive disorders, their relatives and care providers focused more on self‐help issues and the access to adequate care^13^ than our participants did. Nevertheless, the efficacy of treatment and its long‐term success were essential to both samples.

In order to aim for these patient‐derived research topics, funding requirements are one means.^5^, ^27^ When research agendas are set up together with patients, when study objectives are discussed with patients, and when grant applications require a statement on how patients have been and will be involved, researchers are guided to more PI in their studies. During the survey, one patient asked ‘Who is this “research” and when will “it” answer me?’, and called for more and understandable feedback of research results to patients. Thus, we prepared a summary of our results using plain language for publication in the DGZ magazine. Although the current study used indirect involvement,^2^ it is targeted at direct participation of patients into the whole cycles of future research projects.^28^ According to experiences from other countries with a longer tradition in PI, coordination and collaboration across institutions, identification and evaluation of effective PI strategies for divergent contexts, and eventually, a change in the research culture is strongly recommended.^29^

Limitations of the study are firstly attributable to the resources available. The meta‐review was conducted only by one researcher, and priority setting was conducted more economically than proposed for example by the James Lind Alliance.^8^ Other limitations refer to the small sample of rather experienced professionals who were mainly psychologists with a research focus. The sample is characterized by German‐speaking participants. Patients’ mean age was 40, and they were rather well‐educated and predominantly female. Although theoretical saturation was achieved, that is, no new categories emerged during categorization, a larger international sample could help extend the results to other health contexts. For that, the questionnaire is available upon request from the corresponding author.

## 5 CONCLUSION

Since patients are the ‘ultimate recipients’ of psychotherapy research results, their early involvement is especially useful.^28^ In this sense, our study points out one way to involve patients into OCD research. According to the results, commonalities between patients and professionals emerged from ranking the most (treatment gains that persist long‐term) and least (effectiveness of group versus individual psychotherapy) important research goals, and also from the open items (psychotherapy and its efficacy, disease development and maintenance). Professionals viewed most research goals as more important, which may be due to their work and interests. Still, evidence‐based practice provides a framework for the combination of patient preferences, clinical expertise and research results,^30^ and early research involvement of OCD patients may be especially fruitful.

## 6 PRACTICE IMPLICATIONS

Patients and professionals prioritized clinically relevant topics such as psychotherapy and its efficacy or disease development and maintenance for OCD research. Future research should take up these topics and should also further involve patients in the research process. Patients are experts with regard to their disease, and they are the people, research is done for. Therefore, research should focus on what ‘patients feel has most relevance to their lives’.^29^

## CONFLICTS OF INTEREST

None.

7

**Table 3:** Categories inductively derived from open questions on the top 5 research goals

Patient priorities	Indication quotient^a^	Priority quotient^b^
Disease development and maintenance	0.26	0.29
Psychotherapy and its efficacy	0.23	0.23
Course of the disease	0.12	0.11
Psychopharmacotherapy	0.11	0.11
Improving the quality of care	0.08	0.08
Others (therapy‐related questions, self‐help, research criticism)	0.20	0.18

Professionals’ priorities	Indication quotient^a^	Priority quotient^b^
Psychotherapy and its efficacy	0.36	0.42
Optimizing existing therapies	0.17	0.17
Disease development and maintenance	0.15	0.17
Improving the quality of care	0.17	0.13
Others (OCD subtypes, OCD‐related disorders)	0.15	0.11

^a^ (No. of indications/ total indications).

^b^ (No. of inverted priority values/ total inverted priorities); ranges from 0 to 1.

## Data Availability

The data that support the findings of this study are available from the corresponding author upon reasonable request.
