# Supplementary material for: Research priorities set by people with OCD and OCD researchers: Do the commonalities outweigh the differences?
Source: Health Expect. 2019 Nov 28;24(Suppl 1):40–6. doi: 10.1111/hex.13005 (PMC8137491; doi:10.1111/hex.13005)
Supplement: Supplementary file 1 [file HEX-24-40-s002.pdf]

Supplement A. Reviews included in the meta-review.

1. Anholt GE, Kempe P, de Haan E, et al. Cognitive versus behavior therapy: Processes of change in the creatment of obsessive-compulsive disorder. *Psychother Psychosom* 2008;77:38–42. https://doi.org/10.1159/000110058
2. Fisher PL, Wells A. How effective are cognitive and behavioral treatments for obsessive–compulsive disorder? A clinical significance analysis. *Behav Res Ther* 2005;43:1543–58. https://doi.org/10.1016/j.brat.2004.11.007
3. Gava I, Barbui C, Aguglia E, et al. Psychological treatments versus treatment as usual for obsessive compulsive disorder (OCD). *Cochrane Database Syst Rev* 2007;2:CD005333. https://doi.org/10.1002/14651858.CD005333.pub2
4. Hofmann SG, Smits JAJ. Cognitive-behavioral therapy for adult anxiety disorders: A meta-analysis of randomized controlled trials. *J Clin Psychiatry* 2008;69:621-32.
5. Hohagen F, Wahl-Kordon A, Lotz-Rambaldi W, Muche-Borowski C. *S3-Leitlinie Zwangsstörungen*. Berlin Heidelberg: Springer-Verlag; 2014.
6. Ipser JC, Carey P, Dhansay Y, Fakier N, Seedat S, Stein DJ. Pharmacotherapy augmentation strategies in treatment-resistant anxiety disorders. *Cochrane Database Syst Rev* 2006;4:CD005473. https://doi.org/10.1002/14651858.CD005473.pub2
7. Jónsson H, Hougaard E. Group cognitive behavioural therapy for obsessive-compulsive disorder: A systematic review and meta-analysis. *Acta Psychiatr Scand* 2009;119:98-106. https://doi.org/10.1111/j.1600-0447.2008.01270.x
8. Komossa K, Depping AM, Meyer M, Kissling W, Leucht S. Second-generation antipsychotics for obsessive compulsive disorder. *Cochrane Database Syst Rev* 2010;12:CD008141. https://doi.org/10.1002/14651858.CD008141.pub2
9. Krisanaprakornkit T, Sriraj W, Piyavhatkul N, Laopaiboon M. Meditation therapy for anxiety disorders. *Cochrane Database Syst Rev* 2006;1:CD004998. https://doi.org/10.1002/14651858.CD004998.pub2
10. Miyasaka LS, Atallah ÁN, Soares B. Valerian for anxiety disorders. *Cochrane Database Syst Rev* 2006;4:CD004515. https://doi.org/10.1002/14651858.CD004515.pub2
11. Miyasaka LS, Atallah ÁN, Soares B. Passiflora for anxiety disorder. *Cochrane Database Syst Rev* 2007;1:CD004518. https://doi.org/10.1002/14651858.CD004518.pub2.
12. Olthuis JV, Watt MC, Bailey K, Hayden JA, Stewart SH. Therapist-supported Internet cognitive behavioural therapy for anxiety disorders in adults. *Cochrane Database Syst Rev* 2016;3:CD011565. https://doi.org/10.1002/14651858.CD011565.pub2.
13. Ori R, Amos T, Bergman H, Soares-Weiser K, Ipser JC, Stein DJ. Augmentation of cognitive and behavioural therapies (CBT) with d-cycloserine for anxiety and related disorders. *Cochrane Database Syst Rev* 2015;5:CD007803. https://doi.org/10.1002/14651858.CD007803.pub2.
14. Rodriguez-Martin JL, Barbanoj JM, Pérez V, Sacristan M. Transcranial magnetic stimulation for the treatment of obsessive-compulsive disorder. *Cochrane Database Syst Rev* 2003;2: CD003387. https://doi.org/10.1002/14651858.CD003387.
15. Rosa-Alcázar AI, Sánchez-Meca J, Gómez-Conesa A, Marín-Martínez F. Psychological treatment of obsessive-compulsive disorder: A meta-analysis. *Clin Psychol Rev* 2008;28:1310-25. https://doi.org/10.1016/j.cpr.2008.07.001
16. Rothbart R, Amos T, Siegfried N. Pharmacotherapy for trichotillomania. *Cochrane Database Syst Rev* 2013;11:CD007662. https://doi.org/10.1002/14651858.CD007662.pub2.

17. Skapinakis P, Caldwell D, Hollingworth W. A systematic review of the clinical effectiveness and cost-effectiveness of pharmacological and psychological interventions for the management of obsessive-compulsive disorder in children/adolescents and adults. *Health Technol Assess* 2016;20:1-392. https://doi.org/10.3310/hta20430

18. Soomro GM, Altman DG, Rajagopal S, Oakley Browne M. Selective serotonin re-uptake inhibitors (SSRIs) versus placebo for obsessive compulsive disorder (OCD). *Cochrane Database Syst Rev* 2008;1:CD001765. https://doi.org/10.1002/14651858.CD001765.pub3

19. Wu H, Yu D, He Y, Wang J, Xiao Z, Li C. Morita therapy for anxiety disorders in adults. *Cochrane Database Syst Rev* 2015;2:CD008619. https://doi.org/10.1002/14651858.CD008619.pub2
