# Supplementary material for: Research priorities set by people with OCD and OCD researchers: Do the commonalities outweigh the differences?
Source: Health Expect. 2019 Nov 28;24(Suppl 1):40–6. doi: 10.1111/hex.13005 (PMC8137491; doi:10.1111/hex.13005)
Supplement: Supplementary file 2 [file HEX-24-40-s001.pdf]

Supplement B. Category system for the professionals' top 5 research priorities (individual priority given in brackets).

Psychotherapy and its efficacy	No. of indications / total indications	No. of inverted priority values / total inverted priorities
• (1) What treatments are effective for OCD?[E4]<br>• (1) Prediction of effective techniques [E3]<br>• (1) What is an effective psychotherapy (for OCD)?[E4]<br>• (1) How to encourage the patients' motivation for therapy? [E7]<br>• (1) How to encourage the patients' motivation to use confrontation (like exposure)? [E7]<br>• (1) Why do which treatments help people with OCD? [E5]<br>• (1) How to deal with patients who are resistant to standard treatment/ do not benefit from it? [E2]<br>• (1) How to help patients with severe OCD? Which treatment must be offer patients who did not benefit from CBT or medication? [E8]<br>• (2) Differential indication: What patients do benefit from which kind of treatment? [E2]<br>• (2) What is the difference between responders and non-responders? [E5]<br>• (2) Why do obsessive-compulsive symptoms often return even if the treatment has been successful? [E7]<br>• (2) Which specific parts or modules of the treatment were helpful and which were not?[E4]<br>• (3) What are the mechanisms of action in treatment for OCD? / How does therapy for OCD work? [E2]<br>• (4) What is the amount of patients who benefit from self-help books without any therapeutic support? [E1]<br>• (5) Are there new treatments for OCD? [E8]	15/41=.36	62/149=.42
**Optimizing existing therapies**		
• (1) Optimize learning by extinction [E3]<br>• (1) How to enhance the patients benefit from therapeutic offers? [E1]<br>• (2) Develop and improve psychotherapeutic approaches and medical ones as well (e.g. deep brain stimulation) [E8]<br>• (2) Reduce stress during exposure [E1]<br>• (3) Reduce stress in long-term patients who cannot mention concrete concerns anymore [E1]<br>• (4) Efficacy and effectiveness of supporting exposure by virtual reality to simplify its implementation and enhance its availability. [E8]<br>• (4) How to combine medication and psychotherapy optimally?[E7]	7/41=.17	25/149=.17
**Disease development and maintenance**		
• (1) Why do some people contract an OCD but not others? [E5]<br>• (1) What causes OCDs? (especially on neuronal level) [E8]<br>• (1) Why does someone contract an OCD but not an addictive disorder?[E8]<br>• (2) What mechanisms of action cause OCD? [E4]<br>• (2) How do neuro-biological aspects vs. personal experiences affect the development and maintenance of OCDs? [E7]<br>• (3) What is the perceived functionality of obsessive-compulsive symptoms for the patient (e.g. intrapsychic like regulation of emotion,	6/41=.15	26/149=.17

interpersonal)? [E7]		
**Improving the quality of care**		
• (2) In many cases patients have been suffering from OCDs for years. Therefore they've got difficulties in coping with life on their own again. Could living in a therapeutic community be helpful, at least on an interim basis? (It is already available for eating disorder patients) [E8]<br>• (2) Why are there still therapists who don't like to work clinically with OCD or don't do it at all? [E8]<br>• (3) What is the distribution of standard treatment in therapeutic practice? [E2]<br>• (3) Exposure is the gold standard treatment for OCD. Why is this technique used rarely or not at all by a lot of therapists? [E8]<br>• (4) How to improve cooperation and collaboration between physicians, ambulant psychotherapists and clinics to accelerate the receiving of treatment. [E8]<br>• (4) There is still a long latency to receive psychotherapy. How to improve psychological care and therapy for patients? [E8]<br>• (5) Motivation: Why are there some patients with OCD who are not willing to look for support or to undergo treatment? [E8]	7/41=.17	19/149=.13
**Others**		
**OCD subtypes**<br>• (2) Who develops which subtype and why? [E5]<br>• (3) Do treatments have to be adapted for different subtypes and how should it be done? [E5]<br>• (3) How to support patients with different subtypes of OCD optimally[E7]<br>• (5) Are there subtypes? [E2]	6/41=.15	17/149=.11
**OCD-related disorders**<br>• (3) Little research has been done on OCD-related disorders like trichotillomania and skin picking. Are there any neurobiological approaches? Which brain areas are involved or activated differently? Are there any medical imaging procedures? What findings are necessary to improve therapeutic treatment? [E8]<br>• (3) More accurate differentiation of diagnostic criteria for OCD (with ppor insight) and schizophrenia with obsessive-compulsive features[E8]		
