# Supplementary material for: Research priorities set by people with OCD and OCD researchers: Do the commonalities outweigh the differences?
Source: Health Expect. 2019 Nov 28;24(Suppl 1):40–6. doi: 10.1111/hex.13005 (PMC8137491; doi:10.1111/hex.13005)
Supplement: Supplementary file 3 [file HEX-24-40-s003.pdf]

Supplement C. Category system for the patients‘ top 5 research priorities (individual priority given in brackets).

**Psychotherapy and its efficacy**	No. of indications / total indications	No. of inverted priority values / total inverted priorities
• (1) Are there alternatives to CBT or rather confrontation especially for patients who don’t respond to it? [P11]<br>• (1) Why a there just short-term psychotherapeutic treatment for patients with chronic OCD even if it doesn’t depend billing or administrative decisions? I was told it’s to avoid addiction to the therapist. Is it true or does the patient benefit from a long-term treatment? [P34]<br>• (1) What techniques of HRT and exposure are more helpful? [P55]<br>• (1) Is CBT really always the best choice?[P91]<br>• (1) Is deep brain stimulation an effective treatment for OCD? What happens during such a treatment? [P104]<br>• (1) What is the best psychotherapeutic treatment for OCD? [P108]<br>• (1) Is a therapy in groups consisting of patients with similar disorders a more appropriate treatment compared to individual therapy? [P52]<br>• (1) What is the most successful treatment for OCD in regard to its long-term success? [P124]<br>• (1) Why is exposure the only effective part of CBT? [P137]<br>• (1) How to expedite my desensitization? [P110]<br>• (1) How to independently maintain therapy achievements in my daily routine? [P117]<br>• (1) How to reduce paedophiliac obsessive thoughts in patients? [P93]<br>• (1) What percentage of patients (given an optimal treatment) is able to reduce their symptoms to a level they can live a normal life with? Is it even possible to live a life without limitations? [P42]<br>• (1) What kind of therapy has the best outcomes? [116]<br>• (2) Are there alternatives to overrated CBT especially to help all those patients who don’t respond to it? [P22]<br>• (2) So far CBT is still the preferred treatment for OCD. For patients it‘s the only option to choose. Isn’t there a need for new therapeutic concepts to incorporate different approaches like ACT and relaxation techniques? Not only in a clinical setting [P34]<br>• (2) Could deep brain stimulation be a long-term effective treatment for OCD? [P66]<br>• (2) Is inpatient treatment more effective compared to outpatient psychotherapeutic treatment? [P52]<br>• (2) Most of the patients suffer a lot from their behaviour just like addicted persons. So advices to change this behavior are perceived as heartless and cynical. What are really helpful approaches? [P72]<br>• (2) What is a more effective confrontational treatment: to deal with the worst thing that could happen or to be oblivious of nothing won’t happen? [P92]<br>• (2) How to support CBT? Are additional techniques like meditation useful (learn to distance from thoughts, let thoughts come and go)? [P18]<br>• (2) How to maintain the outcomes of a treatment successfully? (follow-up care)[P31]<br>• (2) How do exposure or flooding have to be implemented to ensure best outcomes?[P46]<br>• (2) What aspects of CBT, psychoanalytic treatment and depth psychology have an effect on OCD? Which ones do not? [P67]	44/192=.23	160/690=.23

• (2) Is successful exposure necessarily linked to habituation?[P116]<br>• (2) Treatment for OCD oftentimes seems to be standardized, all OCDs are treated the same. Is it possible to generate individualized therapy concepts in consultation with the patient? (individualized instead of standardized therapeutic treatment, not all washing compulsions are the same) [P40]<br>• (2) Is online therapy an option for patients who want to live their life on in defiance of their psychological strain? (e.g. universitary studies cause the need for relocation so a long-term treatment seems to be all but impossible) [P82]<br>• (2) Are there cases the usefulness of CBT is limited because depth psychological aspects have to be faced first? [P91]<br>• (3) Are there reasonable alternatives to exposure or rather to flooding? [P46]<br>• (3) Isn't long-term inpatient psychotherapy (10 weeks) clearly more effective than a short-term one (5 weeks)? [P71]<br>• (3) To what extend depth psychology, psychoanalytic treatment and CBT can/have to be combined? [P91]<br>• (3) How to explain that classic CBT but IBT and MCT as well are effective treatments for OCD? [P116]<br>• (3) What are options to improve my long-term well-being by myself? (active patient participation in recovery) [P124]<br>• (3) My obsessive thoughts don't induce a feeling of anxiety but a feeling of guilt. Classic exposure doesn't work on this optimally because feelings of guilt are more long-lasting than feelings of anxiety (even though I don't carry them out). As result I feel despondent and depressed. What is an effective technique to deal with this feeling of guilt? [P42]<br>• (4) How helpful are mindfulness-based interventions as a treatment for OCD? [P22]<br>• (4) get rid of magical thinking [P44]<br>• (4) What is the importance of information of family and friends about OCD to succeed in psychotherapeutic treatment? [P52]<br>• (4) Is there given enough assistance and supervision by CB-therapists especially during exposure? [P91]<br>• (4) How to cure this disease? [P135]<br>• (4) Isn't there clearly a bigger benefit from inpatient treatment with two sessions per week instead of one? (particularly with regard to the first and last session cannot be considered) [P71]<br>• (5) Are there skills used in case of emergency to deal with an acute crisis? (like for borderline personality disorder) [P40]<br>• (5) Is depth psychology helpful as a treatment for OCD? [P54]<br>• (5) What different treatments are effective for OCD? [P104<br>• (5) What are the advantages of oupatient treatment over inpatient treatment? [P116]		
**Disease development and maintenance**		
• (1) What causes OCD? What would a psychotherapy look like when it's adjusted to that causes?[P22]<br>• (1) What causes the development of OCD and how to counteract it best?[P58]<br>• (1) Where does OCD come from? Is there a physical cause? [P17]<br>• (1) What is the current state of research on causes of OCD? [P21]<br>• (1) Is there a link between OCDs and hormone diseases? (thyroid disease, sex hormone diseases in women, PMS etc.) [P31]<br>• (1) What happens on physical level? [P37]<br>• (1) More (intense) research should be done on causes of OCD. There are lots of approaches (neurobiological, psychoanalytical,…) to	50/192=.26	199/690=.29

consider. [P40]<br>• (1) Research on causes – Are there neurobiological links (e.g. on genetic level)? Are there personal experiences that provoke or affect OCD? [P45]<br>• (1) Reasons for OCD [P49]<br>• (1) neurobiological and genetic background of OCD [P57]<br>• (1) Why does OCD develop? [P66]<br>• (1) Is there potentially a key moment previous to OCD? [P75]<br>• (1) Are there verifiably biological or rather genetic factors linked to OCD? If so which ones and how to prevent disease outbreak prematurely? [P92]<br>• (1) Are there specific patterns of behaviour or personality traits which point to the development of OCD? How to decrease thr risk of disease outbreak? [P105]<br>• (1) What happens in the brain while development and maintenance of obsessive thoughts? [P114]<br>• (1) Why do I have so many obsessions? [P125]<br>• (1) What specific reasons cause the development of OCD? [P135]<br>• (1) Which bio-chemical processes cause the malfunction of my cerebral metabolism? What brain areas are activated while development and maintenance of OCD compared to healthy person? My CB-therapist only told me about involvement of the limbic system to explain how anxiety increases and decreases. To me it seems to be important to understand what happens in my brain to counteract it by my cognitive and emotional abilities and rationality. To me it seems like specific experiences, habits, adaptation of my parents' behaviour, the environment, external influences, genetic disposition etc. cannot cause my OCD on their own. [P136]<br>• (1) Is there evidence for a negative impact of the endocrine system? [P75]<br>• (1) How does OCD develop (causes and trigger)? [P139]<br>• (1) How to change attitudes and aspects of personality causing development and maintenance of OCD? [P145]<br>• (1) Is OCD treatable? [P152]<br>• (1) Can OCD be cured completely? [P158]<br>• (2) Does it make sense to search for causes of OCD and to work on it? May it be an option to cure OCD? [P21]<br>• (2) What circumstances may lead to OCD? [P37]<br>• (2) What are causes for OCD? [P54]<br>• (2) Do changes in brain matter? (I just saw a TV show on VOX, apparently there are even brain surgery and use of deep brain stimulation.) [P58]<br>• (2) What causes the development of OCD? How to involve approaches of developmental psychology to answer this question? [P114]<br>• (2) What causes my OCD?[P125]<br>• (2) Is OCD hereditary? [P135]<br>• (2) Is there a link between personality of an individuum and development of an OCD? Are there personal characteristics which promote the maintenance or even the development of OCD (e.g. hypersensitivity etc.)? [P136]<br>• (2) OCD with comorbidity ADHD [P57]<br>• (2) What genetic factors / environmental conditions are respponsible for OCD?[P139]<br>• (2) What attitudes and aspects of personality stimulate development and maintenance of OCD? [P145]		

• (2) How fast can I count on improvement of my symptomatology? [P152]<br>• (3) Will research ever figure out how OCD develops and how to counteract? [P25]<br>• (3) Are there different causes for OCD? Someone has a strong genetic predisposition to OCD, another one's OCD is strongly linked to comorbidity. [P40]<br>• (3) What (stressful) life events do trigger OCD? [P58]<br>• (3) Why does an OCD develop but not another disease? Which conditions are obligatory and which are additional ones? [P67]<br>• (3) What are causes of OCD? Are there any new findings? [P95]<br>• (3) To which extend nurture and stressful life events are relevant in development of an OCD? [P105]<br>• (3) Why am I not able to defeat my OCD by reason of rationality? I feel like I'm at the mercy of my brain oftentimes. [P136]<br>• (3) What is the link between OCD and anxiety? Is OCD linked to depression? [P114]<br>• (4) What factors promote OCD outbreak? What factors can cure or control it in general? [P60]<br>• (4) Everybody knows the experience of checking your door to be locked when you leave. How does normal behavior develop to an OCD? [P66]<br>• (4) Why do some obsessive thoughts lead to compulsive behavior but others not? [P82]<br>• (4) Are there cultural differences in appearance and incidence of OCD? If so why do they develop? [P67]<br>• (4) What is the link between OCD and tics? Is there similarity to tourett syndrome? [P130]<br>• (4) Wie entsteht eine Zwangsstörung? How do OCDs develop?[152]<br>• (5) I experienced my OCD to be linked to my feelings of safety and control. How is the intensity of OCD linked to feelings of safety and control? [P130]<br>• (5) What happens in the brain during OCD? [P145]		
**Course of disease**		
• (1) Can one ever be cured completely? [P10]<br>• (1) Why do obsessive thoughts reoccur after symptom-free time for years? [P14]<br>• (1) Which resiliences facilitate a positive course of the disease (coping strategies) [P18]<br>• (1) Can one ever be again like before OCD? Meaning completely symptom-free and not even the chance for relapse. [P25]<br>• (1) reaching a state of being symptom-free [P44]<br>• (1) How high are the chances for recovery (completely without symptoms, if not possible "acceptable" without symptoms) [P46]<br>• (1) Is OCD with obsessive thoughts completely curable? [P54]<br>• (1) Is healing considering my long disability still possible? I have OCD since 1980 consistently. [P95]<br>• (2) Why can't I recognize them directly (in case of relapse) as what they are: obsessive thoughts. Instead I feel bad again and many things (luckily not everything) starts again in my head. [P14]<br>• (2) Can cognitive biases regarding my often exaggerated evaluation of risk and responsibility be undone in a way that I think "normally" or are they partly irreversible? [P42]<br>• (2) decrease relapses [P44]<br>• (3) Preventing OCD symtoms [P44]<br>• (3) Will I ever be able to live without medication? [P54]	22/192=.11	76/690=.11

• (3) Why does my OCD get even worse? [P125]<br>• (3) Which reasons/factors contribute to „relapses“?[P145]<br>• (3) What is the relapse rate (relapse in old behavior patterns) after psychotherapy? [P52]<br>• (4) How can the relapse rate be decreased? Why do OCD symptoms come back again?[P11]<br>• (4) Are there better opportunities to prevent relapses? [P25]<br>• (4) What actions can prevent relapses best? [P145]<br>• (5) How does OCD develop in the course of decades? [P22]<br>• (5) What is the percentage of „healed“ OCD patients after psychotherapy? [P52]<br>• (5) Will my suffering get worse and end up in suicide? [P125]		
**Psychopharmacology**		
• (1) Can one figure out which medication will be suited for which patient? Talking about “individual medicine”. [P71]<br>• (1) I would appreciate research on effective medication against inner anxiety and disturbance, which are present in OCD patients at any time. Giving highly dosed antidepressants may slightly mitigate symptoms. I’m treated this way for 10 years and by the time there are irreversible side effects, anxiety and strong compulsive acts stay. Benzodiazepine offers me help in extreme situations. [P140]<br>• (1) What exactly are the biochemical associations? What role do genetics, metabolism and nutrition play? Where are potential targets for new agents? [P106]<br>• (1) Is there medication which works like CBT and which is as effective without needing CBT? [P82]<br>• (1) Effective medication and treatment methods are missing. [P17]<br>• (2) When will there be psychotropic drugs which don’t promote weight gain? [P25]<br>• (2) research about pharmaceutic therapy, alternatives to SRI; medication which doesn’t promote weight gain [P11]<br>• (2) Intensification of pharmaceutic research [P27]<br>• (2) Is it helpful to check serotonin levels before medication take in? Can conclusions be drawn on, if medication will be helpful? [P71]<br>• (2) Are there better medications now with less adverse side effects, e.g. weight gain? [P95]<br>• (2) Which herbal or orthomedical agents truly work (e. g. amber, holy thistle, …)[P106]<br>• (3) Will there be well-tolerated medication, which will help getting over OCD symptoms? [P21]<br>• (3) Are there other medications than SSRI or antipsychotics, which could help, maybe something about a missing proteine, which was in the media [P22]<br>• (3) Which nutriets / medication (neurotransmitter precursors) promote the reduction of activity surplus incl. the resulting cognitive deficits considering the body’s total energy expenditure? Discrepance between glucose needs brain and sceletal muscles -> increased stress -> changes in energy balance with tendency to eating disorder/ obesity [P55]<br>• (3) Could specific medication for OCD treatment be developed? [P66]<br>• (3) How does medication help? [P152]<br>• (3) What about the long-time safety of the already found effective medication? [P106]<br>• (4) Which medication qualifies for OCD patients, who cannot oder don’t want to take SSRI? [P40]<br>• (4) Will there be better medication anytime soon? [P54]	23/192=.12	77/690=.11

• (4) Why does no medication help against OCD? [P125]<br>• (4) Can individual pharmacotherapy be developed based on exact diagnostics and the patient's targets? [P106]<br>• (5) Over which time period do OCD patients take medication? A medicament like SSRI should be a temporary help for no more than three years, especially because of its adverse effects. In practice patients take their medication often over decades. [P71]<br>• (5) How can the for the specific patient most effective antidepressant be found before the treatment starts? [P106]		
**Improving the quality of care**		
• (1) There aren't enough statutory health insurance therapists. Waiting time til treatment is one year or longer. Many don't record any patients on their waiting lists anymore because waiting times would be unacceptable. In comparison to physical diseases one doesn't feel taken seriously because of these waiting time. [P17]<br>• (1) Expertise of more psychiatrists and psychologists [P27]<br>• (1) Where are similarly affected people of my age in my region? [P60]<br>• (1) How can a therapist deal with bizarre behaviors (even if they occur unappealing), without showing this? [P154]<br>• (2) Why are there so few therapists, who are experts for OCD? [P20]<br>• (2) How does therapy proceed? Which alternatives are possible for me as a child/ teenager/ pupil/ adult/ pensioner in my region? [P60]<br>• (2) Is there/ How can an adequate occupational rehabilitation after treatment be realized? [P104]<br>• (2) Wie können Patienten bestmöglich über ihre Erkrankung aufgeklärt werden? How can patients be educated about their disease best possible?[124]<br>• (2) How can OCD affected people be reached, if they can't reach out to treatment offers? [P154]<br>• (3) Why are there still CBT therapists, who aren't using exposition or who don't offer treatment for OCD patients? [P31]<br>• (3) How can refusal of OCD treatment on the therapist side be diminished?[P154]<br>• (4) How can it be possible that affected people get help at an early stage? [P104]<br>• (4) Why is there still a treatment deficit for OCD (few experts, long waiting times)? [P116]<br>• (5) Are there physicians with professional expertise in my region, where I can get help, if needed? [P60]<br>• (5) Why is there such little information about OCD in the German speaking area? [P152]	15/192=.08	52/690=.08
**Others**		
RESEARCH-RELATED<br>• (1) Why does research on this topic barely show progress? [P15]<br>• (1) Who is this „research" and when does it answer my questions? 99,9% of these studies seem to be Diploma/Bachelor/Master studies, about which "results" the participants are never informed. [P43]<br>• (2) Why is there so little research about OCD? [P15]<br>• (3) Why is there so little research about OCD? [P20]<br>• (5) Are there any unimportant research questions? [P14]<br>• (5) Why is there so little research about OCD? [P20]	38/192=.20	126/690=.18

SELF-HELP

- (1) What does effective self-help look like?[P20]
- (3) self-help programmes for affected people [P11]
- (3) What „help“ exists for me (medication, SHG, ...) [P60]
- (4) How can I openly deal with my disease and where is it reasonable not to expose oneself to unnecessary stigmatization? [P124]
- (5) How can I deal with guilt feelings concerning my disease? [P124]

THERAPY-RELATED

- (1) Why is the power of OCD still underrated? [P72]
- (1) What does the self-confidence of the patient look like? [P75]
- (1) How developed is the sensitivity of the patient?[P75]
- (1) How well can a patient look at himself from the outside? [P75]
- (1) If the cause for a certain symptom lies in a boundary crossing of a particular person, could the symptoms better/ eliminate if the patient speaks to the person, who caused the symptoms, and draws the line throughout the conversation? [P130]
- (1) Aren’t CBT and exposition too rude and forced methods? To make it more comprehensive, an example: birth in hospital. The mother experiences herself as powerless and never gets the chance to guide this natural process herself with only getting medical help, if needed. I see OCD against its pathologization as a natural thing. I don’t define it as a disease but as a warning signal that something in life isn’t balanced anymore. If one goes through OCD with dignity, something enriching can evolve (back to the birth example: having labors are obsessional thoughts/ irrational anxieties). [P138]
- (1) What happens to the core of OCD-problems, if OCD is simply “off-treated” by exposition (without working on the problem behind)? [P67]
- (2) How well can the patient process conflicts? [P75]
- (2) Could hypnosis be an effective treatment method? [P105]
- (2) Alternative treatment methods – involving subcounsciousness, e.g. hypnosis. [P45]
- (2) Since a couple of years I wake up with an earworm. Reason for this was a hard relationship and through this I had obsessional rumination every morning. These earworms replaced my ruminations, in that way that my last dream is accompanied by a song, by which I wake up. My question: What is the association between earworms/ catchy songs and OCD/ obsessional rumination? [P130]
- (2) Association between hypersensitivity, intellectual giftedness and some kinds of OCD. [P138]
- (2) How can I increase my focus on objectivity – I feel like this brain areal is less developed. [P110]
- (3) What influence does nutrition have on OCD? What influence do coffee and alcohol have? [P14]
- (3) Is it possible to train concrete action commands in order to interrupt compulsive rituals? STOP isn’t working well. [P34]
- (3) How can it be possible to give society an understanding of OCD? This is urgently required. [P104]
- (3) Does the patient have structure in his daily life? How does this structure look like? [P75]
- (3) To what extent is sport important in the treatment of OCD? [P82]
- (3) How can I continuously live my self-confidence? …how to diminish insecurities and anxieties… [P110]
- (3) My mother is extremely critical. I have OCD because of her behavior. Unfortunately new symptoms occur still today when I visit her,

which doesn’t happen often, because she criticizes something/ crosses a boundary. I fight back but still have new symptoms. What can one do concretely in order not to get more symptoms from a person, from whom one knows that one gets new symptoms from (besides restricting contact and defending onseself)? [P130]<br>• (3) How strongly is OCD connected with self-confidence? [P135]<br>• (4) How well can the patient isolate himself from his environment? [P75]<br>• (4) Should one exercise brain (and body) more that obsessive thoughts cannot spread? [P14]<br>• (4) What role does fantasy play in the context of OCD? [P114]<br>• (4) Why is this disorder so badly portrayed in the media? Why is it ridiculed? [P20]<br>• (5) Is the patient a loner? How well does he get along within groups? How is he adapted? [P75]<br>• (5) relieve anxieties [P44]		
